# Bovine Leptospirosis in Caatinga Biome, Brazil: New Insights into Diagnosis and Epidemiology

**DOI:** 10.3390/tropicalmed8030177

**Published:** 2023-03-17

**Authors:** Nathanael Natércio da Costa Barnabé, Rafael Rodrigues Soares, Deivyson Kelvis Silva Barros, Denise Batista Nogueira, Flávia Teresa Ribeiro da Costa, João Pessoa Araújo Júnior, Camila Dantas Malossi, Leila Sabrina Ullmann, Diego Figueiredo da Costa, Maria Luana Cristiny Rodrigues Silva, Severino Silvano dos Santos Higino, Carolina de Sousa Américo Batista Santos, Sérgio Santos de Azevedo, Clebert José Alves

**Affiliations:** 1 Academic Unit of Veterinary Medicine (UAMV), Federal University of Campina Grande (UFCG), Patos 58708-110, Brazil; 2 Faculty of Veterinary Medicine and Zootechny (FMVZ), University of São Paulo (USP), São Paulo 05508-220, Brazil; 3 Institute of Biosciences, Department of Microbiology and Immunology, University of the São Paulo State (UNESP), Botucatu 18618-687, Brazil; 4 Department of Veterinary Science, Federal University of Paraíba (UFPB), Areia 58397-000, Brazil

**Keywords:** *Leptospira* spp., serology, cut-off point, bacteriological culture, PCR, semiarid conditions

## Abstract

Bovine leptospirosis causes economic losses and raises public health concerns. It is possible that there are peculiarities in the epidemiology of leptospirosis in regions with a semiarid climate, such as the Caatinga biome in Brazil, where the climate is hot and dry, and the etiological agent require alternative routes of transmission. This study aimed to close knowledge gaps to the diagnosis and epidemiology of *Leptospira* spp. infection in cows from the Caatinga biome, Brazil. Samples of the blood, urinary tract (urine, bladder and kidney) and reproductive tract (vaginal fluid, uterus, uterine tube, ovary and placenta) were collected from 42 slaughtered cows. Diagnostic tests included were the microscopic agglutination test (MAT), polymerase chain reaction (PCR) and bacterial isolation. Anti-*Leptospira* spp. antibodies were found in 27 (64.3%) of the animals analyzed using MAT at a 1:50 dilution (cut-off 50), while 31 (73.8%) animals had at least one organ/fluid where the presence of *Leptospira* spp. DNA was identified, and 29 animals (69%) were positive at bacteriological culture. The highest sensitivity values for MAT were obtained at the cut-off point of 50. In conclusion, even under hot and dry climate conditions, it is possible that *Leptospira* spp. can spread through alternative routes such as venereal transmission; moreover, a cut-off of 50 is recommended for the serological diagnosis of cattle from the Caatinga biome.

## 1. Introduction

Leptospirosis is widespread globally and is among the diseases that impair livestock production rates. It raises public health concerns because of its zoonotic impact and that, even after more than a century of research, the vaccines available do not confer lasting immunity or cross-protection between serovars [1]. The agent, pathogenic *Leptospira* species, has several hosts, and exposure ensues through direct contact with infected animals or indirectly via water and soil contaminated with urine [2,3]. Transmission can also occur through contact with the vaginal fluid and placental remains, copulation and also vertically [4]. The losses in livestock production result from abortions, stillbirth, weak offspring, diminished growth rate, diminished milk production or agalactia and death [5]. In cattle, this disease is more associated with subfertility and early embryonic death when the serovars is host-adapted, such as serovar Hardjo [6]. Studies conducted in the semiarid region of Brazil have suggested that both domestic [7,8,9,10,11,12] and wild animals [13,14] present high frequencies of *Leptospira* spp. This occurs despite the hot and dry climate conditions of this region, which are adverse to the survival of this bacterium in the environment. 

Among the methods for diagnosing leptospirosis, there are indirect methods that investigate the immune response of the host and direct methods that target the detection of leptospires or their DNA. The microscopic agglutination test (MAT) has been recommended as a serological test by the World Organization for Animal Health (OIE) because of its capacity to demonstrate the most likely infecting serogroup [15]. Moreover, polymerase chain reaction (PCR) is a fast technique with high sensitivity and specificity and is, therefore, very reliable [16,17], although microbiological isolation is considered to be the gold standard [18,19]. The paired use of these diagnostic methods increases the capacity for detecting a positive animal in order to cover all situations where leptospires are not shed in urine, or chronic phase where antibody titers can be low; in addition, microbiological culture and isolation is still the only way to confirm the autochthonous *Leptospira* spp. serovars.

Given that there are differences in incidence related to climatic conditions [20] and to individual adaptation to the agent, such that they remain serologically unidentified, the test protocol that fits best needs to be established so as to ensure greater diagnostic accuracy. Sheep maintained in semiarid conditions have shown resistance to *Leptospira* spp. as well as short seroconversion periods [21,22]. Furthermore, surveys have found that the MAT had better performance with a cut-off point of 50, considering PCR and bacteriological culturing as gold standards [10,12]. Thus, the selection of an appropriate cut-off point for serology is relevant to increasing sensitivity and avoiding false-negative results. However, surveys focusing on improving the serology cut-off points applied to the diagnosis of cattle leptospirosis are lacking concerning Brazilian semiarid regions. 

Low rainfall and high temperatures characterize the semiarid region of Brazil, and when associated with the peculiarities of the existing vegetation, the Caatinga, a biome exclusive to Northeastern Brazil with abundant wild fauna—such as tamanduá-mirim (*Tamandua tetradactyla*), preá (*Cavia aperea*), mocó (*Kerodon rupestris*) and cachorro-do-mato (*Cerdocyon thous*), offers unique epidemiological conditions that can influence the occurrence of infectious diseases such as leptospirosis. Therefore, this study aimed to evaluate the serological, molecular and microbiological methods applied to the diagnosis of leptospirosis in cows from the Caatinga biome in Northeastern Brazil.

## 2. Materials and Methods

### 2.1. Study Area

Biological samples were collected from the public slaughterhouse of Patos county (latitude: 7°00′19″ south; longitude: 37°16′48″ west) in the state of Paraíba, Northeastern Brazil. The animals originated from areas located in the Caatinga biome and were slaughtered within a maximum period of two days. This biome is an exclusively Brazilian biome and has a semiarid climate characterized by long periods of water scarcity and stunted vegetation. The climate is hot and dry, with a rainy season in summer/autumn and rainfall concentrated between March and April. However, precipitation may occur at any time between January and May. Droughts can last for more than a year, resulting in a negative water balance plus high solar radiation [23,24]. The period during which the present study was conducted corresponded to the dry season, with average rainfall and temperature of 0.47 mm and 29.28 °C, respectively [25].

### 2.2. Sampling

The minimum sample size was determined using the following formula for proportion analysis [26]:

$$n=\frac{p_{0}\times q_{0}\times {\left(Z_{1-\beta }+Z_{\frac{\alpha }{2}}\times \sqrt{\frac{p_{1}\times q_{1}}{p_{0}\times q_{0}}}\right)}^{2}}{\left(p_{1}-p_{0}\right)}$$

where

*n* = minimum sample size

Z_α/2_ = 1.96 (Z value for 95% confidence level)

Z_1−β_ = 1.64 (Z value for power of 95%)

*P*_0_ = 33% (reference proportion for PCR positivity) [27]

*P*_1_ = 61.40% (estimate for the experimental proportion of positivity in PCR) [28]

*q*_0_ = 1−*p*_0_

*q*_1_ = 1−*p*_1_

According to these parameters, a minimum of 37 animals were needed to assess true prevalence within a confidence interval of 95%; however, 42 cattle were used. They were all healthy female, aged greater than or equal to 24 months, cross-bred and had no history of vaccination against leptospirosis. According to the data taken from animal movement forms held by the State Veterinary Service of Paraíba, these female cattle came from rural farms located in the semiarid region, from municipalities belonging to two federative units: Paraíba (Cacimba de Areia, Condado, Olho D’Água, Patos, Pombal, Santa Terezinha, São José de Espinharas, São José do Bonfim and São Mamede) and Pernambuco (Buíque, Capoeiras and Exu).

### 2.3. Biological Sample Collection

The blood samples were collected from jugular vein using 8 mL labeled sterile tubes containing a coagulation activator just prior to slaughter of the animals. After collection, the tubes were sent to the laboratory, where they were centrifuged at 1512 g for 10 min, and the serum samples were stored in microtubes at −20 °C.

The bladder, kidney, ovary, uterine tube, uterus and placenta (placentome region) were collected for the direct detection of *Leptospira* spp. The tissues were immediately fragmented by using autoclaved surgical materials and sterile slides for each tissue. After that, the fragments were immediately transferred to a specific room in the slaughterhouse, where there was a Bunsen burner, and then they were deposited onto autoclaved Petri dishes while avoiding contact between the fragments. The material was fragmented into smaller portions of ≈ 2 gm (in duplicate), placed into DNA and RNA-free microtubes and stored at −20 °C for molecular detection and bacteriological isolation. Sterile swabs were used to collect vaginal fluid directly from the cervical–vaginal region, and urine was taken by cystocentesis during evisceration, using 5 mL sterile syringes. Both materials were also stored in duplicates in DNA- and RNA-free microtubes with 0.5 mL of phosphate-buffered saline [10].

### 2.4. Microscopic Agglutination Test (MAT)

The microscopic agglutination test (MAT) was used to detect anti-*Leptospira* spp. antibodies using a collection of 24 serovars belonging to 17 different pathogenic serogroups of five species provided by the Laboratory of Veterinary Bacteriology of the Fluminense Federal University, Niterói, Rio de Janeiro, Brazil, originating from the Pasteur Institute, France. The *Leptospira* species and serovars were *L. interrogans* serovars Copenhageni, Canicola, Autumnalis, Wolffi, Hardjoprajitno, Icterohaemorrhagiae, Pomona, Kennewicki, Hebdomadis, Pyrogenes, Bratislava and Australis; *L. santarosai* seorovars Guaricura, Shermani and Canalzoni; *L. borgpetersenii* serovars Javanica, Tarassovi, Ballum, Mini and Castellonis; *L. kirschneri* serovars Grippotyphosa and Cynopteri; *L. noguchi* serovars Panama and Lousiana [15]. The sera were screened at a 1:50 ratio, and the positive ones were two-fold diluted, in which the final result was the respective highest titer achieved, making use of the ranking technique [16].

### 2.5. Microbiological Culture

Immediately after the collection, three drops of urine, the swab with vaginal fluid and approximately 2 gm of each tissue were inoculated into tubes containing 5 mL of liquid EMJH medium (Difco, BD Franklin Lakes, NJ, USA) with amphotericin B (0.05 mg/mL), 5-fluorouracil (0.1 mg/mL), fosfomycin (0.4 mg/mL), trimethoprim (0.2 mg/mL) and sulfamethoxazole (0.4 mg/mL) [17]. After 24 h, 1 mL of the primary culture was inoculated into a semi-solid EMJH medium without antibiotics, and then incubated in a biological oxygen demand incubator (BOD) at 30 °C. Regardless of the presence of Dinger ring zone, the tubes were examined weekly for 12 weeks using dark-field microscopy.

### 2.6. Leptospira *spp.* DNA Detection and Sequencing

The Dneasy Blood and Tissue Kit (Qiagen, Hilden, Germany) was used to extract the DNA from the urine, vaginal fluid and tissues, as well as vaginal fluid and urine EMJH cultures that exhibited the growth of leptospires under dark-field microscopy examination, according to the manufacturer’s recommendations. The gene *LipL*32, specific to pathogenic leptospires and, therefore, of public health importance, was amplified with *LipL*32-45F (5′-AAG CAT TAC CGC TTG TGG TG-3′) and *LipL*32-286R (5′-GAA CTC CCA TTT CAG CGA TT-3′) primers [29] following the procedures for polymerase chain reaction (PCR) previously described [18]. Primers were used in a concentration of 0.6 μM, 1.0 U Taq polymerase, 2.4 μM MgCl2, 0.3 mM dNTP in a final volume of 25 μL. One cycle of initial denaturation at 94 °C for two minutes, followed by 35 cycles of denaturation at 94 °C for 30 s, annealing the primers to 53 °C for 30 s and one minute extension with 72 °C and final extension cycle at 72 °C for five minutes were used. PCR products were developed by 2% ultrapure agarose gel electrophoresis stained with Evans Blue (Thermo Fisher Scientific, Waltham, MA, USA) and 100 bp ladder, and DNA bands (≅260 bp) were visualized under ultraviolet light. Strain *Leptospira interrogans* serovar Copenhageni, Fiocruz L1-130 (ATCC BAA-1198) was used as positive control, and ultrapure water was used as a negative control.

*LipL*32-45F and *LipL*32-286R primers [29] were used in the sequencing reactions with the Sequencing Kit Big Dye Terminator v3.1 (Applied Biosystems, Foster City, CA, USA). 3130xl Genetic Analyzer and POP-7 polymer [30] were used for capillary electrophoresis, sequence alignment was conducted by using BioEdit [31], and the dataset strings were obtained from GenBank (National Biotechnology Information Center, Bethesda, MD, USA) (http://www.ncbi.nlm.nih.gov, accessed on 19 November 2022) using the BLAST tool (http://www.ncbi.nlm.nih.gov/BLAST/, accessed on 19 November 2022). SeaView4 software [32] was applied during the phylogenetic analysis, and the neighbor’s association model was used to build a phylogenetic tree with a bootstrap value of 1000 repetitions (http://tree.bio.ed.ac.uk/software/figtree/, accessed on 26 November 2022), as viewed through the FigTree v1.4.3 (http://tree.bio.ed.ac.uk/, accessed on 26 November 2022). The phylogenetic reconstruction included *Leptospira* sequences for comparison.

### 2.7. Statistical Analysis

The proportions of positive animals and samples were compared by using the chi-squared test with Yates’ continuity correction or Fisher’s exact test using the BioEstat 5.3 software [33] with a 5% significance level (*p* ≤ 0.05). The sensitivity and specificity of MAT at the cut-off points of 50, 100, 200 and 400 were calculated using DAG_Stat software [34], deeming the PCR and microbiological culture singly results as gold standards.

## 3. Results

### 3.1. Leptospira *spp.* Antibody Detection

Twenty-seven out of 42 animals (64.3%; 95% CI = 49.2–77%) presented with anti-*Leptospira* spp. antibodies at the cut-off point of 50, and Sejroe, Tarassovi, Australis, Ballum, Djasiman and Hebdomadis were the reactive serogroups. At the cut-off of 100, 16 animals (38.1%; 95% CI = 25–53.2%) were seroreactive for Sejroe, Tarassovi and Hebdomadis serogroups, and at titer of 200, 10 animals (23.8%; 95% CI = 10.9–36.7%) reacted for the serogroups of Sejroe, Tarassovi and Hebdomadis, and eight animals (19%; 95% CI = 10–33.3%) were seroreactive for Sejroe, Tarassovi and Hebdomadis at the titer of 400. Overall, the most frequent serogroups were Sejroe (55.6%) and Tarassovi (22.2%), with titers ranging from 50 to 1600 (Table 1), considering only the seroreactive animal (n = 27).

### 3.2. Leptospira *spp.* Molecular Results

Leptospiral DNA was found in at least one sample in 31 animals (73.8%; 95% CI = 58.9–84.7%). Among the 309 samples, PCR detected leptospires DNA in 90 (29.1%). The most frequent PCR positive samples were the placenta (13 samples: 86.7%), uterus (17 samples: 40.5%) and kidneys (14 samples: 33.3%). There were statistically significant differences (*p* < 0.05) between urine and kidney, urine and uterus, uterus and uterine tube, and the placenta differed from all of the biological materials (Table 2).

### 3.3. Microbiological Culture

In microbiological culture, the pathogen was visualized in at least one sample in 29 animals (69%; 95% CI = 54–80.9%), and 73 cultures (23.6%) out of 309 were found to be positive, highlighting the placenta (10 samples: 66.7%), uterus (13 samples: 31%), ovary (11 samples: 26.2%) and kidneys (11 samples: 26.2%). Urine differed statistically (*p* < 0.05) from the kidney, uterus and ovary, and the placenta differed from all of the biological materials (Table 2). *Leptospira* spp. DNA was identified in at least one microbiological culture in 19 animals (45.3%; 95% CI = 30.2–60.3%), and the biological materials with the highest frequencies were uterus (eight samples: 19.1%) and placenta (two samples: 13.3%).

### 3.4. Leptospira *spp.* DNA Sequencing

**Figure 1:**
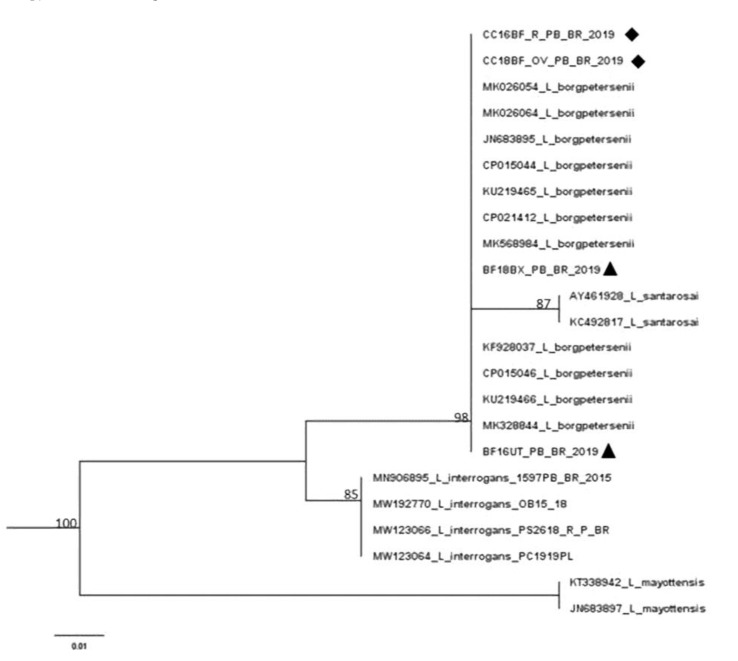
A phylogenetic tree based on the nucleotide sequence alignment of the *LipL32* gene of *Leptospira* spp. constructed using the neighbor-joining method with 1000 replicates. ▲ ♦ Samples sequenced from tissues. Samples sequenced from cultures.

Due to budget issues, DNA sequencing from the PCR products was possible in two samples taken from tissues (bladder and uterus) and two from cultures (kidney and ovary) from different animals. These samples showed a 99% similarity with *Leptospira borgpetersenii* (Figure 1).

### 3.5. Performance of Diagnostic Tests

Overall, the molecular test showed good performance in diagnosing *Leptospira* spp. infection, with high sensitivity (100.00%) and specificity (84.6%) deeming microbiological culture results as gold standards, especially for detecting leptospiral DNA from the uterine tube, with 100% sensitivity and specificity (Table 3). Table 4 and Table 5 show the sensitivity and specificity of the MAT for the different cut-off points (50, 100, 200 and 400) based on using the PCR or microbiological culture results as gold standards. It was verified that, regardless of the biological material used and the gold standard, the highest sensitivity values in the MAT were obtained for the titer of 50.

## 4. Discussion

The high frequency of seroreactivity found (64.3%) indicates that, even under adverse climatic conditions, *Leptospira* spp. can be present in herds in the Caatinga biome of Brazil. There was variation in the serogroups found (Sejroe, Tarassovi, Australis, Ballum, Djasiman and Hebdomadis), suggesting that there are different sources of infection, although Sejroe (55.6%) and Tarassovi (22.2%) were more prevalent. In other investigations conducted in semiarid regions, Sejroe (58.17%) was the most prevalent serogroup, followed by Icterohaemorrhagiae (17.32%) and Australis (4.58%) [7], as well as Sejroe (36.8%), Hebdomadis (26.3%), Australis (10.5%), Djasiman (10.5%), Ballum (5.3%) and Pomona (5.3%) [35]. Surveys of different regions of Brazil have demonstrated that regardless of the biome involved, Sejroe was most prevalent: 50.68% average prevalence in northeastern region [7,35,36,37]; 72.65% in northern region [38,39]; 30.3% in central-western region [40]; 47.01% in southeastern region [41,42,43,44]; and 48.56% in southern region [45,46]. The plurality of types of *Leptospira* suggests that contact with other animals exists, but there is the possibility that cattle without signs and symptoms of *Leptospira* spp. infection may carry and spread other strains within the species (adaptive process) [47].

The contrast between unfavorable climatic conditions (average rainfall and temperature of 0.47 mm and 29.28 °C, respectively, in the dry season) and a significant proportion of seroreactive animals, especially regarding the Sejroe serogroup, provides evidence for intraspecies transmission, which is less dependent on environmental factors because cattle are maintenance hosts [5,48]. Tarassovi is one of the main serogroups found in cattle [49]; however, there have been few reports of it in Brazil [44]. The Australis serogroup is associated with pigs and causes reproductive failure, abortion, stillbirth, fetal mummification and weak piglets [50]; it has also been described in mammals maintained in zoos [51]. The reservoirs of the serogroup Ballum are rodents, especially mice (*Mus musculus*). Asymptomatic mice harbor bacterium in their kidneys, which makes them an important source of infection for humans or animals [50]. The confirmation of the presence of this serogroup in cattle suggests that rodents have had access to cattle food, which usually consists of protein concentrate stored to ensure supplementation during periods in which there is low production of phytomass, or the rodents have had access to the facilities and feeders/water troughs.

PCR data can generate difficulties in interpreting the interrelation of results, as some factors, such as the amount of bacterial DNA in the sample and its quality, may not be favorable [38,52]. Leptospiral DNA was detected in 73.8% of the animals, but the true frequency may be higher, as negativity can also be correlated with DNA concentrations below the detectable threshold [53]. When comparing the positivity rates between different biological materials, there were statistically significant differences (*p* < 0.05) that demonstrated that leptospires accumulate preferentially in the placenta, uterus and kidneys when compared to the urine. This can be explained by the fact that the size, morphology (spirochete/helicoidal) and translational motility of the agent facilitate access to these organs, allowing it to escape the immune system due to the physical barrier that prevents contact between antibody and antigen molecules. The broad presence of leptospires in the reproductive tract reinforces that this is a site of extra renal bacterial colonization in cattle, which can act as adapted hosts, as has already been elucidated in other studies [28,54].

When analyzed from an epidemiological perspective, it is clear that, especially in the Caatinga biome, where climatic conditions are adverse for bacterial survival in the environment, extra renal colonization by leptospires is a strong indication that, in this region, venereal transmission and vertical transmission of leptospires are alternative routes in contrast to transmission through the urine, in which the external environment is part of the epidemiological cycle. The PCR results confirmed that, for animals maintained under field conditions, as well as urine, vaginal fluid could be valuable for identifying carriers both in slaughterhouses and farms.

*Leptospira* spp. was isolated from 29 animals (69%). By comparing the positivity rates between the different materials, there were statistically significant differences (*p* < 0.05) that demonstrated the tropism of the microorganism for the placenta, uterus, kidney and ovary, while the frequency of occurrence in the urine was lower, as explained earlier. The results from the cultures revealed that working on microbiological isolation, using tissue from the placenta, uterus, kidney and ovary can increase the chances of success. However, considering that access to tissue from the uterus, kidney and ovary is difficult, the collection of such material is appropriate in postmortem cases. Leptospires-specific DNA was identified in 19 cultures, among which there was one kidney sample and one ovary sample from different animals. The isolation and characterization of autochthonous strains are important with regard to understanding epidemiology and refining diagnostic tools. Moreover, this introduces the possibility of discovering new species and/or variants. If these circulating strains can be incorporated into vaccines, greater protection for animals in the region can be provided [55].

The sequenced DNA from four samples demonstrated 99% similarity with *L. borgpetersenii*, which belongs to the pathogenic clade and, according to virulence, to subgroup 2, along with the species *L. santarosai*, *L. mayottensis*, *L. weilii* and *L. alexanderi* [1]. *L. borgpetersenii* causes early embryonic loss and estrus repetition, resulting from uterine inflammation and damage caused by embryo invasion [6]. The identification of this species through genetic sequencing provided yet another indication of venereal transmission, and perhaps this pathway was responsible for influencing the high frequency of positive findings during the dry period because any other route that depends on external variables is less likely to succeed. In addition, this species has been reported to infect humans in other countries [56,57,58,59,60,61,62].

Out of the 31 positive animals on PCR, 15 showed positivity in both the reproductive and urinary tracts, 13 were positive only in the reproductive tract and three only in the urinary tract. Animals with urogenital tract involvement potentiate the diffusion of the agent, especially in the rainy season of a semiarid region, due to the possibility of simultaneous transmission via the urinary, venereal and congenital/transplacental routes. The occurrence of a bovine genital leptospirosis syndrome has been proposed [6], and it is an infection dissociated from renal/systemic leptospire colonization, which is very important in the dry period when the bacterium only has a short survival window in the environment. Therefore, in drought seasons, the urinary tract may be less relevant to the dissemination of *Leptospira* spp., except in microclimates, which would have been unlikely in the present study, considering that the animals were living on several different farms.

Under the conditions of the Caatinga biome, molecular detection proved to be reliable for diagnosing pathogenic *Leptospira* spp. infection and showed high sensitivity (100%) and specificity (84.6%). This reinforces the importance of molecular methods, and many factors contribute to this accuracy, e.g., the extraction kit, thermostable DNA polymerase, laboratory equipment and operating procedure. Reports of leptospiral DNA detection in animals negative upon MAT are common, and if the animal presents with a high antibody titer, the outcome of PCR tends to be negative. The high sensitivity of PCR often eliminates the need for isolation as a confirmatory result, and it is suitable for emergency situations, given that it is fast and enables early diagnosis based on blood analysis during leptospiremia [29].

The findings of this research reinforce the hypothesis that, although very useful for herd diagnosis, serology may be an insufficient tool to identify carriers individually, thus making it necessary to detect the presence of the agent to identify and safely treat the carriers [10]. Therefore, PCR proved to be a great diagnostic alternative because it is fast and has high sensitivity and specificity. The higher proportion of animals positive for PCR compared to MAT is due to the fact that, in some cases, depending on the serovar–host interaction and individual host responses, leptospires have low antigenicity and, in addition, the infection may be in the chronic phase [48]. Thus, a seronegative animal is not always free from infection. The analysis of the cut-off points for serology showed that, regardless of the biological material investigated, the highest sensitivity values of MAT were obtained at a titer of 50, deeming both PCR and microbiological culture as gold standards. In cattle from Colombia [63], a titer of 50 presented 95% and 89% sensitivity and specificity, respectively, in comparison to microbiological culture; however, the authors only used urine samples for the direct diagnosis of *Leptospira* spp. infection. Similar results were also found by our research group in sheep from Brazilian semiarid regions [10,12].

It is worth mentioning that the diagnostic tests for *Leptospira* spp. infection are imperfect, and the appropriate combination of methods and specimens for each stage of infection improves diagnostic accuracy. The principle behind MAT is simple, but it requires maintaining a panel of live leptospires, representing a biological risk and restricting its practice to specialized laboratories [64]. In PCR, some biological materials require proper treatment to avoid inhibitory substances [65]; in addition, it is limited by its inability to identify specific strains [54]. Microbiological confirmation is laborious and time-consuming [43]; in addition, contamination by secondary microorganisms’ compromises culture performance [44].

Our data show that a cut-off point of 50 may be more suitable for cattle from the Caatinga biome, and, in this scenario, high sensitivity values are essential to reduce the number of false-negative results upon MAT. In addition, given the results of this survey, cut-off points below 50 should also be considered as they are relevant in other species, such as sheep and swine. This is very important from epidemiological and infection control points of view as it avoids the maintenance of infected animals in the herd since subclinical infections are very common [19].

It is important to emphasize that although the recommendation by the World Organization for Animal Health (OIE) of the use of cut-off point of 100 for the serological diagnosis of leptospirosis in cattle [15], this statement has limitations regarding the detection of renal and genital Leptospira spp. carriers, which generally have low antibody titers [48]. In addition, the use of a lower cut-off point than the conventionally used was based on the question whether this methodology demonstrates favorable results only for small ruminants [10,22] or also for cattle, being both in semiarid conditions. The central idea is to assess whether increasing the sensitivity of the MAT increases the correlation between serological results and Leptospira spp. carrier condition.

## 5. Conclusions

This study provides important information relating to the diagnosis and epidemiology of *Leptospira* spp. infection in cattle from the Caatinga biome of Brazil. The results indicate that, even under the adverse environmental conditions of the Brazilian Caatinga biome, leptospires may survive and propagate through alternative routes of transmission, such as sexual pathways, and the high proportion of PCR-positive cows in the genital tract highlights the possible role of females in venereal transmission. Moreover, a cut-off of 50 should be considered for the serological diagnosis of cattle from the Caatinga biome. The study also shows that cows are commonly exposed to leptospires in the Caatinga biome, and this constitutes a One Health-based concern, demonstrating the importance of broad studies where large numbers of humans and animals coexist when investigating zoonotic infections and when planning and implementing control measures for cattle-associated leptospirosis.

## Figures and Tables

**Table 1:** Predominant serogroups and respective antibody titers at MAT in cattle from the Caatinga biome, Brazil.

Serogroups	Titers *						Total (%)
	50	100	200	400	800	1600	
Sejroe	3	5	1	2	2	2	15 (55.6)
Tarassovi	3	1	1	-	-	1	6 (22.2)
Australis	2	-	-	-	-	-	2 (7.4)
Ballum	2	-	-	-	-	-	2 (7.4)
Djasiman	1	-	-	-	-	-	1 (3.7)
Hebdomadis	-	-	-	1	-	-	1 (3.7)
Total (%)	11 (40.7)	6 (22.2)	2 (7.4)	3 (11.1)	2 (7.4)	3 (11.1)	27 (100)

* Only the highest antibody titer was counted.

**Table 2:** *Leptospira* spp. molecular and microbiological diagnoses according to different types of biological material from cattle from the Caatinga biome, Brazil.

Sample	Total	PCR		Culture	PCR of Culture	
		31 */42 (73.8%)		29 */42 (69%)	13 */42 (31%)	
		+ (%)	Sequencing	+ (%)	+ (%)	Sequencing
Urine	42	6 (14.3) ^a^	-	4 (9.5) ^a^	1 (2.4)	-
Bladder	42	10 (23.8) ^ab^	1	8 (19.1) ^ab^	1 (2.4)	-
Kidney	42	14 (33.3) ^b^	-	11 (26.2) ^b^	2 (4.8)	1
Vaginal fluid	42	10 (23.8) ^ab^	-	9 (21.4) ^ab^	1 (2.4)	-
Uterus	42	17 (40.5) ^b^	1	13 (31) ^b^	8 (19.1)	-
Uterine tube	42	7 (16.7) ^a^	-	7 (16.7) ^ab^	2 (4.8)	-
Ovary	42	13 (31) ^ab^	-	11 (26.2) ^b^	2 (4.8)	1
Placenta	15	13 (86.7) ^c^	-	10 (66.7) ^c^	2 (13.3)	-
Total	309	90 (29.1)	2	73 (23.6)	19 (6.2)	2

* = number of positive animals; + = positive samples. Different lowercase letters in the same column indicate significantly different proportions (*p* ≤ 0.05).

**Table 3:** Performance of molecular tests for diagnosing *Leptospira* spp. infection in cattle from the Caatinga biome, Brazil, based on the microbiological result as the gold standard.

Biological Material	Microbiological Culture		PCR			
	29 */42 (69.05%)		31 */42 (73.81%)			
	+	-	+	-	SEN	SPE
Urine	4	25	6	25	100	94.7
Bladder	8	21	10	21	100	94.1
Kidney	11	18	14	17	100	90.3
Vaginal fluid	9	20	10	21	100	97
Uterus	13	16	17	14	100	86.2
Uterine tube	7	22	7	24	100	100
Ovary	11	18	13	18	100	93.6
Placenta	10	5	13	2	100	40
Total	29	13	31	11	100	84.6

* = number of positive animals; + = positive samples; - = negative samples; SEN = sensitivity (%); SPE = specificity (%).

**Table 4:** Performance of different MAT cut-offs using molecular analysis (PCR) as gold standard per biological material.

Biological Material	Antibody Titers (Cut-Off)															
	50				100				200				400			
	27 */42 (64.3%)				16 */42 (38.1%)				10 */42 (23.8%)				8 */42 (19%)			
	PCR		MAT		PCR		MAT		PCR		MAT		PCR		MAT	
	+	-	SEN	SPE	+	-	SEN	SPE	+	-	SEN	SPE	+	-	SEN	SPE
Urine	4	23	66.7	36.1	3	13	50	63.9	2	8	33.3	77.8	2	6	33.3	83.3
Bladder	7	20	70	37.5	5	11	50	65.6	4	6	40	81.3	3	5	30	84.4
Kidney	9	18	64.3	35.7	6	10	42.9	64.3	5	5	35.7	82.1	4	4	28.6	85.7
Vaginal fluid	6	21	60	34.4	5	11	50	65.6	3	7	30	78.1	3	5	30	84.4
Uterus	8	19	47.1	24	6	10	35.3	60	4	6	23.5	76	4	4	23.5	84
Uterine tube	4	23	57.1	34.3	1	15	14.3	57.1	1	9	14.3	74.3	1	7	14.3	80
Ovary	8	19	61.5	34.5	3	13	23.1	55.2	3	7	23.1	75.9	3	5	23.1	82.8
Placenta	7	2	53.9	0	5	1	38.5	50	3	1	23.1	50	3	1	23.1	50

* = number of MAT positive animals at MAT; + = positive samples; - = negative samples; SEN = sensitivity (%); SPE = specificity (%).

**Table 5:** Performance of different MAT cut-offs using microbiological culture as gold standard per biological material.

Biological Material	Antibody Titers (Cut-Off)															
	50				100				200				400			
	27 */42 (64.3%)				16 */42 (38.1%)				10 */42 (23.8%)				8 */42 (19%)			
	MC		MAT		MC		MAT		MC		MAT		MC		MAT	
	+	-	SEN	SPE	+	-	SEN	SPE	+	-	SEN	SPE	+	-	SEN	SPE
Urine	4	23	100	39.5	3	13	75	65.8	2	8	50	79	2	6	50	84.2
Bladder	6	21	75	38.2	4	12	50	64.7	3	7	37.5	79.4	3	5	37.5	85.3
Kidney	8	19	72	38.7	5	11	45.5	64.5	5	5	45.5	83.9	4	4	36.4	87.1
Vaginal fluid	6	21	66.7	36.4	5	11	55.6	66.7	3	7	33.3	78.8	3	5	33.3	84.9
Uterus	7	20	53.9	31	5	11	38.5	62.1	3	7	23.1	75.9	3	5	23.1	82.8
Uterine tube	4	23	57.1	34.3	1	15	14.3	57.1	1	9	14.3	74.3	1	7	14.3	80
Ovary	7	20	63.6	35.5	3	13	27.3	58.1	3	7	27.3	77.4	3	5	27.3	83.9
Placenta	6	3	60	40	4	2	40	60	2	2	20	60	2	2	20	60

* = number of positive animals at MAT; + = positive samples; - = negative samples; SEN = sensitivity; SPE = specificity; MC = microbiological culture.

## Data Availability

The datasets generated during and/or analyzed during the current study are available from the corresponding author upon request.
